# *In Vitro* Assessment of the Antibacterial Potential of Silver Nano-Coatings on Cotton Gauzes for Prevention of Wound Infections

**DOI:** 10.3390/ma9060411

**Published:** 2016-05-25

**Authors:** Federica Paladini, Cinzia Di Franco, Angelica Panico, Gaetano Scamarcio, Alessandro Sannino, Mauro Pollini

**Affiliations:** 1 Department of Engineering for Innovation, University of Salento, Via per Monteroni, Lecce 73100, Italy; federica.paladini@unisalento.it (F.P.); angelica.panico@unisalento.it (A.P.); alessandro.sannino@unisalento.it (A.S.); 2 CNR-IFN U.O.S. Bari, Via Amendola 173, Bari 70126, Italy; cinzia.difranco@uniba.it (C.D.F.); gaetano.scamarcio@uniba.it (G.S.); 3 Dipartimento Interateneo di Fisica, University of Bari Aldo Moro, Via Amendola 173, Bari 70126, Italy

**Keywords:** silver dressing, biofilm, antibacterial, wound infection

## Abstract

Multidrug-resistant organisms are increasingly implicated in acute and chronic wound infections, thus compromising the chance of therapeutic options. The resistance to conventional antibiotics demonstrated by some bacterial strains has encouraged new approaches for the prevention of infections in wounds and burns, among them the use of silver compounds and nanocrystalline silver. Recently, silver wound dressings have become widely accepted in wound healing centers and are commercially available. In this work, novel antibacterial wound dressings have been developed through a silver deposition technology based on the photochemical synthesis of silver nanoparticles. The devices obtained are completely natural and the silver coatings are characterized by an excellent adhesion without the use of any binder. The silver-treated cotton gauzes were characterized through scanning electron microscopy (SEM) and thermo-gravimetric analysis (TGA) in order to verify the distribution and the dimension of the silver particles on the cotton fibers. The effectiveness of the silver-treated gauzes in reducing the bacterial growth and biofilm proliferation has been demonstrated through agar diffusion tests, bacterial enumeration test, biofilm quantification tests, fluorescence and SEM microscopy. Moreover, potential cytotoxicity of the silver coating was evaluated through 3-[4,5-dimethylthiazol-2-yl]-2,5-diphenyltetrazolium bromide colorimetric assay (MTT) and the extract method on fibroblasts and keratinocytes. Inductively coupled plasma mass spectrometry (ICP-MS) was performed in order to determine the silver release in different media and to relate the results to the biological characterization. All the results obtained were compared with plain gauzes as a negative control, as well as gauzes treated with a higher silver percentage as a positive control.

## 1. Introduction

Wound healing can be delayed by a number of factors, such as wound colonization by microorganisms and infections. Many microorganisms in a wound produce factors detrimental to healing, such as toxins and enzymes [1]. In burn wounds, bacterial infections can frequently occur because of the accumulation of dead tissues, compromised immune system and blood supply [2,3]. In chronic wounds, bacteria persist in an adhesive matrix biofilm more resistant to antimicrobial therapy [4,5]. Multidrug-resistant organisms are increasingly implicated in both acute and chronic wound infections, thus compromising the chance of therapeutic options [6].

To prevent infection or critical colonization of wounds, the choices are locally or systemically delivered antibiotics, or dressings containing topical antimicrobial agents [7,8]. The intrinsic resistance of bacterial cell within biofilm to conventional antimicrobials has encouraged new approaches for the treatment of biofilm-associated infections; among them, the use of silver preparations represents an interesting route towards new antimicrobials. Indeed, unlike antibiotics, silver interferes with multiple components of bacterial cell structure and functions, making it less affected by specific micro-environmental variations [9,10,11]. Moreover, in the form of nanoparticles, silver offers many advantages in wound care and nanoparticles can also overcome existing drug resistance mechanisms [12].

The anti-inflammatory effect of nanocrystalline silver offers exciting new applications [13]. In addition to the use of silver sulfadiazine as a topical cream, more contemporary studies have explored the use of silver-impregnated dressings to manage infections [7]. Silver plays an important role in the management of burn wounds by reducing the microbial growth within a wound dressing and wound bed. The antimicrobial properties of silver-impregnated wound dressings against Gram-positive and Gram-negative bacteria have been demonstrated in several *in vivo* studies [14,15,16].

Burn wounds treated with silver nanoparticles demonstrated better cosmetic appearance and scarless healing [17]. In contrast to topical silver agents, wound dressings containing silver as an antimicrobial agent have been recently introduced in various designs by the wound care industry [14]. An important advantage of using silver nanoparticles in wound dressings is the continuous release of silver ions and possibility of coating the device on both the outer and inner sides, thus enhancing the antimicrobial efficacy [12].

In this work, silver-treated wound dressings have been developed for the prevention of bacterial infections in wounds and skin injuries. Traditional cotton gauzes were deposited with silver nanoparticles on both sides by adopting a deposition technology based on the *in situ* photo-reduction of silver nitrate. The efficacy of the adopted technology in providing a homogeneous coating of the cotton fibers has been verified through scanning electron microscopy (SEM) and thermo-gravimetric analysis (TGA). The antibacterial effectiveness of the silver-treated gauzes was tested in comparison with positive and negative controls. The inhibition to bacterial growth and proliferation induced by the presence of silver nanoparticles was verified through agar diffusion tests and bacterial enumeration on *Staphylococcus aureus*. The efficacy in inhibiting the bacterial biofilm formation and proliferation on the silver treated wound dressing was also demonstrated by biofilm quantification tests, fluorescence and scanning electron microscopy. The biocompatibility of the silver coating was verified through MTT assay on fibroblasts and keratinocytes and the results were related to ICP-MS analysis for evaluation of silver release in different media.

## 2. Experimental Section

### 2.1. Deposition of Silver Nanoparticles on Cotton Gauzes

Sterile cotton gauzes (10 × 10 cm, count 12/8) purchased by a local pharmacy were selected as textile substrates because of their wide use as traditional dressings in the management of wounds and burns. Coatings of silver nanoparticles were deposited on the gauzes through the *in situ* photo-reduction of silver nitrate [18] by adopting a technology described for different natural and synthetic materials [19,20,21,22]. In general, the technology involves three definite steps, namely the preparation of the impregnating silver solution, the deposition of the silver solution onto the surface of the material, and the UV exposure (365 nm) of the wet substrate. The process parameters such as the composition of the silver solution, the deposition method and the UV exposure time are defined as functions of the specific nature and application of the materials. In the specific case of cotton gauze for wound dressing application, the substrates were dip coated in an alcoholic silver solution for 1 min and then immediately exposed to ultraviolet irradiation (365 nm, 500 W, distance 20 cm) in order to induce the photo-chemical deposition of silver nanoparticles on the surface of the cotton fibers. Particularly, in this study an impregnating silver solution containing 0.5 w/v % of silver nitrate dissolved in methanol was adopted for the production of the experimental samples. Methanol was adopted as both reducing agent and solvent, even if deionized water can also be added to reduce the costs of the treatment. Moreover, samples treated with 4 w/v % Ag were also produced and adopted as positive control, whilst plain gauzes were used as negative control. After the silver treatment, the samples were carefully washed in deionized water and dried in an oven at 60 °C for 2 h.

### 2.2. SEM Analysis on Silver Coated Gauzes

The morphological characterization was performed by a field emission scanning electron microscope (FE-SEM), mod. ∑igma Zeiss (Jena, Germany). The samples were firstly coated with a 2 nm palladium layer by an electron beam evaporator to avoid charging and examined at a 2–15 kV acceleration voltage, 30 µm aperture, in top-view. To map the actual surface of the samples, the in-lens detector was used, revealing the secondary electrons generated in the upper range of the interaction bulb and therefore containing direct information on the sample surface morphology.

### 2.3. Thermo-Gravimetric Analysis TGA

The amount of silver deposited on the cotton substrates was calculated through thermo-gravimetric analysis TGA (Mettler, Columbus, OH, USA). Untreated and silver treated samples were heated from room temperature to 1000 °C in nitrogen flow and with a heating rate of 10 °C/min. The percentage of silver deposited on the gauze was calculated as the difference between the solid residues obtained from the treated and the untreated samples. Each type of sample was tested in triplicate and the results were expressed as mean values ± standard deviation.

### 2.4. Qualitative and Quantitative Evaluation of the Antibacterial Capability

The efficacy of the silver-treated samples in reducing the bacterial viability and proliferation was evaluated by agar diffusion tests and bacterial enumeration on *S. aureus* SA1 mucoid in comparison with the untreated sample.

The agar diffusion tests were conducted according to Standard ‘SNV 195920-1992’. The procedure consisted in incubating the samples for 24 h at 37 °C in contact with bacteria on nutrient agar plates and then in evaluating the presence of an area of inhibited bacteria growth around the samples. The antibacterial capability of the sample was defined as a function of the width of the inhibition area, according to the levels provided by the Standard. Thus, if the width of the bacterial inhibition area is greater than 1 mm, a “good” antibacterial activity can be associated with the sample; on the other hand, if the sample is fully covered by bacteria, its antibacterial activity is labelled as “insufficient”.

The bacterial enumeration was performed through the serial dilution method. Samples of untreated and silver treated cotton gauzes were incubated overnight at 37 °C in nutrient agar inoculated with 100 μL of *S. aureus* suspension (inoculating cell density 0.94 × 10^6^ CFU/mL). After incubation, the samples were removed from the broth, and serial dilutions were performed in sterile phosphate buffered saline. From each dilution, 100 μL of the solution were extracted and plated in triplicate on nutrient agar plates. After incubation at 37 °C for 24 h, the bacterial colonies grown on the agar plates were counted and the average number of colonies was calculated for each sample. The samples were tested in triplicate. In order to evaluate the effect of the silver coating on the bacteria viability, the results were expressed as average percentages of bacterial proliferation calculated with respect to the initial concentration of the bacterial suspension.

### 2.5. Quantification of Bacterial Biofilm on Cotton Gauzes

Samples of untreated and silver treated cotton gauzes (1 cm^2^) were placed in a 24-well microtiter and incubated overnight at 37 °C with 2 mL nutrient broth inoculated with *S. aureus* (inoculating cell density 0.94 × 10^6^ CFU/mL). The samples were washed three times with phosphate-buffered saline to remove the non-adherent bacteria from the surface and then transferred to a sterile falcon containing 2 mL phosphate buffered saline. Then, the samples were vortexed for 2 min to detach the adherent bacteria and incubated at 37 °C for 4 h. For quantification of total number of bacteria, in triplicate, aliquots of suspension were analyzed with a spectrophotometer (Visible spectrophotometer V-1200, VWR, Radnor, PA, USA) and the optical density (OD600 nm) was reported as bacterial proliferation expressed in colony-forming unit (CFU/mL) (1 OD600 = 1.5 × 10^8^ CFU/mL) [23].

Moreover, in order to evaluate the bacterial viability and to assess the bactericidal effect of the silver treated samples, the plate count method was also adopted. The aliquots of suspension were diluted in phosphate buffered saline through serial dilutions and then 100 μL of each dilution were spread on nutrient agar plates. The plates were incubated at 37 °C for 24 h and bacterial colonies grown on the agar plates were counted. The average number of colonies and the log reduction were calculated for each sample, with respect to the untreated sample.

### 2.6. Fluorescence Microscopy on Adherent Bacteria

Another set of samples was incubated overnight at 37 °C in a 24-well microtiter, in the same experimental conditions described in the previous section. After incubation, the samples were removed from the multi-wells, washed three times with phosphate buffered saline and transferred to other sterile wells for further SEM analysis. The wells used for gauzes incubation were washed with phosphate-buffered saline (PBS) to remove any non-adherent bacteria and then analyzed. The biofilm maturated on both gauzes and multi-wells surface was stained using green-fluorescent nucleic acid stain (SYTO9, Molecular Probes, Eugene, OR, USA). After 15 min dark incubation, bacteria were analyzed through a Zeiss inverted microscope (Axio Vert. A1 FL-LED, Oberkochen, Germany).

### 2.7. SEM Analysis of the Bacterial Adhesion on Cotton Gauzes

Samples of untreated and silver-coated cotton gauzes (2 × 2 cm, average weight 15 mg) were UV sterilized for 15 min on each side and incubated overnight at 37 °C in 3 mL of nutrient agar inoculated with 100 µL of *Staphylococcus aureus* suspension (inoculating cell density 0.94 × 10^6^ CFU/mL). After incubation, the samples were washed with phosphate buffered saline in order to remove the non-adherent cells, whilst the adherent bacteria were fixed by using 2.5% glutaraldehyde and 2% paraformaldehyde in cacodylate buffer 0.1 M for one hour. After fixation, the samples were washed three times with cacodylate buffer for 10 min and then dehydrated in serially increasing concentrations of ethanol (25%, 50%, 75% and 100%). Each wash lasted for fifteen minutes. The samples were stored at −20 °C and conditioned at room temperature before SEM analysis.

### 2.8. Cytotoxicity Test

Mouse embryonic fibroblasts 3T3 and human keratinocytes HaCaT were cultured in Dulbecco’s modified Eagle’s medium (DMEM) supplemented with 10% fetal bovine serum (FBS) and antibiotics (1% UI/mL Streptomycin-Penicillin). Cell viability was analyzed through 3-[4,5-dimethylthiazol-2-yl]-2,5-diphenyltetrazolium bromide colorimetric assay (MTT) and the extract method, by adopting the only DMEM as positive control and the cells incubated without samples as negative control. The cells were plated at 1 × 10^5^ cells/mL in 24-well plates and incubated for 24 h in a humidified incubator with 5% CO_2_ at 37 °C. For extract preparation, 0.2 g of untreated and silver treated samples were immersed in 10 mL of phosphate buffer saline and incubated at 37 °C for 24 h. The extracts were sterilized by using 0.22 µm filters and pH close to 7.4 was measured. Then, the extracts were added to 24-wells plates with DMEM.

After incubation for 24 h, the culture medium containing the extracts was removed and 0.5 mg/mL of MTT of DMEM was added to each well and the plates were incubated in a CO_2_ incubator for 2 h. After incubation, the intracellular formazan crystals were solubilized with 1 mL of isoprapanol ad centrifuged at 13,000 *g* for 5 min. The absorbance was determined at 550 nm using a spectrophotometer (VWR V1200). The cell viability percentage was calculated in comparison to the control group obtained without any extract. All the assays were performed in triplicate.

### 2.9. ICP-MS Analysis

Silver release from silver-treated cotton gauzes was calculated through inductively coupled plasma mass spectrometry ICP-MS (iCAP Q, Thermo Scientific, Waltham, MA, USA) in static conditions. Silver treated and untreated samples (0.03 g) were immersed, in triplicate, in deionized water, in phosphate-buffered saline (PBS) and in Dulbecco’s Modified Eagle’s Medium (DMEM) (5 mL) and incubated 24 h at 37 °C. Different media have been adopted in this characterization in order to evaluate possible interactions of the silver coating with chemical compounds, such as salts and amino acids, in comparison with deionized water. After the incubation time, an aliquot portion (25 µL) of the different media was diluted with nitric acid 1% (v/v). The samples were analysed, using silver solutions with known concentration as standards (Sigma Aldrich, St. Louis, MO, USA, Silver Standard for ICP, 1000 mg/L).

## 3. Results

### 3.1. Deposition of Silver Nanoparticles on Cotton Gauzes

The silver deposition technology based on *in situ* photo-reduction reaction has been adopted in this work to treat the experimental sample with 0.5 wt/v % silver solution and the control sample with 4 wt/v % silver solution.

### 3.2. SEM Analysis on Silver Coated Gauzes

**Figure 1:**
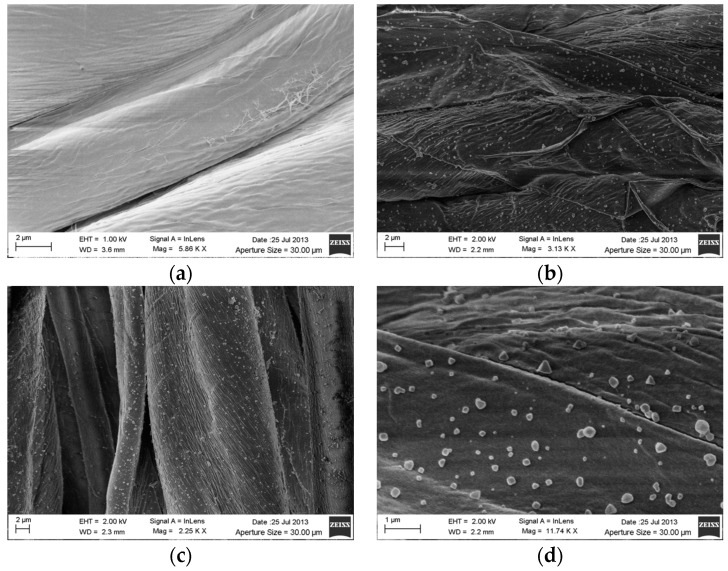
SEM analysis of distribution and dimension of the silver nanoparticles on the cotton fibres: (**a**) neat cotton fibers; (**b**) cotton fibers treated with 0.5 wt/v % silver; (**c**) cotton fibers treated with 4 wt/v % silver; (**d**) cotton fibers treated with 0.5 wt/v % silver at higher magnifications (×11740) for the evaluation of the dimension of the nanoparticles.

The silver treated gauzes and the plain gauze were analyzed by scanning electron microscopy SEM in order to verify the distribution of the silver particles on the cotton fibers, the differences between the silver treated samples as function of the silver concentration tested and the dimension of the silver nanoparticles. The results of the SEM analysis are reported in Figure 1, where an excellent coverage of the fibers is visible for both the silver-treated samples (Figure 1b,c).

SEM analysis at higher magnification (×11740) on the sample treated with 0.5% Ag is reported in Figure 1d, where silver particles with dimensions ranging between about 100 and 300 nm can be observed.

### 3.3. Thermo-Gravimetric Analysis TGA

Thermo-gravimetric analysis TGA was performed in order to quantify the amount of silver deposited on the cotton gauzes as the difference between the solid residues of the silver treated sample and the untreated sample. The samples were tested in triplicate and the percentages of silver deposited resulted 0.29 ± 0.02 wt % and 2.18 ± 0.04 wt % for the sample treated with 0.5 wt/v % and 4 wt/v % respectively.

### 3.4. Qualitative and Quantitative Evaluation of the Antibacterial Capability

**Figure 2:**
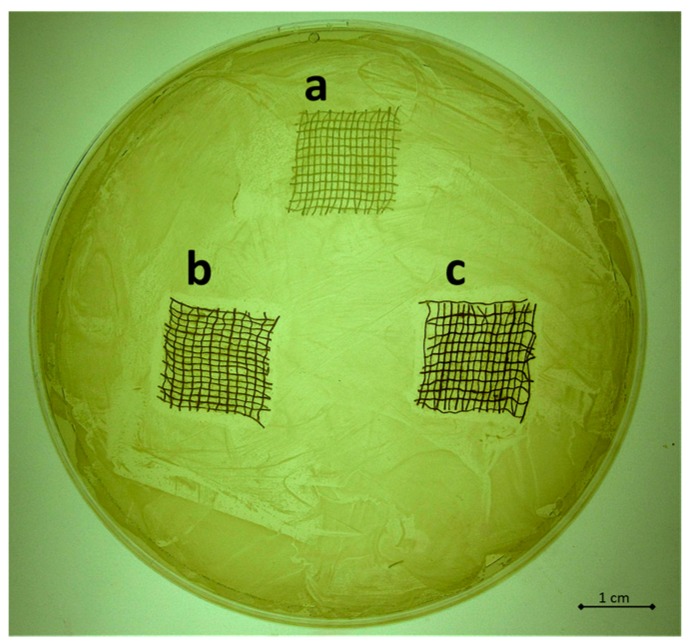
Agar diffusion tests on *S. aureus*: (**a**) untreated sample; (**b**) sample treated with 0.5 wt/v % silver; (**c**) sample treated with 4 wt/v % silver. The presence of the inhibition zone to bacterial growth is clearly visible around both the silver-treated samples.

**Figure 3:**
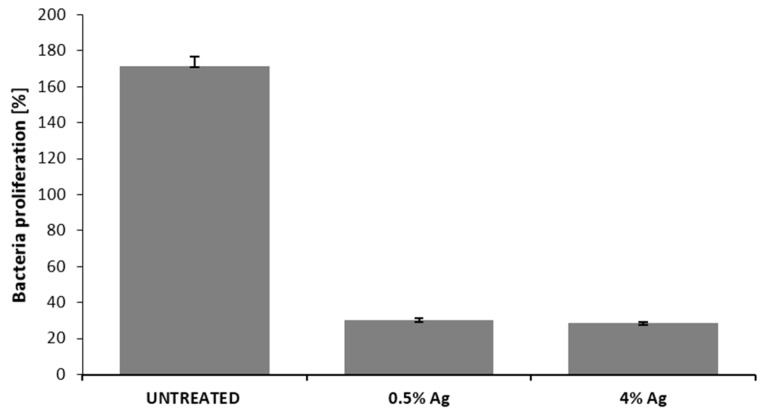
Bacterial proliferation expressed as percentage induced by the silver treated samples in comparison with the plain cotton gauze.

The antibacterial capability of the silver treated samples was tested on *S. aureus* SA1 mucoid through agar diffusion tests and bacterial enumeration. The results obtained by the agar diffusion tests are reported in Figure 2a–c for the untreated sample (Figure 2a), for the sample treated with 0.5 wt/v % Ag (Figure 2b) and for the sample treated with 4 wt/v % Ag (Figure 2c). As clearly visible by comparing the width of the bacterial inhibition growth areas induced by the silver treated samples, no significant difference in the antibacterial properties can be observed between the different concentrations of silver. These data were confirmed by the results obtained by the bacterial enumeration reported in Figure 3, where the percentages of bacterial proliferation calculated corresponded to 31% and 28% for sample treated with 0.5 wt/v % Ag and 4 wt/v % Ag respectively. The untreated sample resulted in a bacterial proliferation of 172%.

### 3.5. Quantification of Bacterial Biofilm on Cotton Gauzes

**Figure 4:**
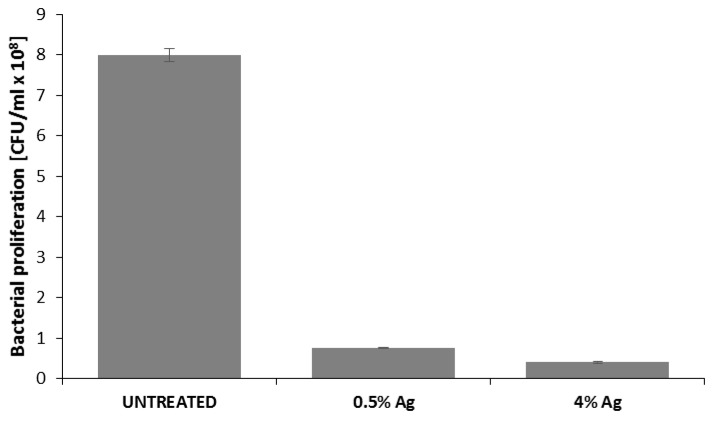
Quantification of bacterial biofilm on cotton gauzes through optical density measurements.

The bacterial biofilm was quantified through both optical density measurements and the serial dilution method. The first technique allows the estimation of total number of bacteria, while the second one is related to live cells only. The results of spectrophotometric analysis are reported in Figure 4 as bacteria proliferation, indicating a 1 log reduction in bacteria adhered to the surface of the silver treated cotton gauzes. The results obtained by the serial dilutions method are reported in Table 1. In comparison with the untreated sample, >3 log reduction was obtained by experimental sample (0.5 wt/v %), and even higher by the control sample (4 wt/v %).

### 3.6. Fluorescence Microscopy on Adherent Bacteria

**Figure 5:**
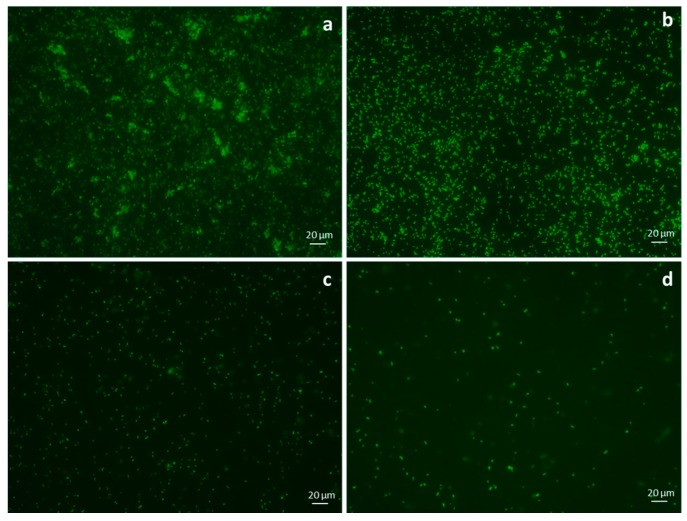
(**a**) Fluorescence microscopy on bacteria adhered on the multi-well plates (magnification ×40) after incubation with no sample; (**b**) untreated sample; (**c**) sample treated with 0.5 wt/v % silver; (**d**) sample treated with 4 wt/v % silver.

The results obtained by fluorescence microscopy on bacteria adhered on the multi-well surfaces are reported in Figure 5a–d for control (Figure 5a), untreated sample (Figure 5b), sample treated with 0.5% Ag (Figure 5c) and sample treated with 4% Ag (Figure 5d). Control sample refers to bacteria adhered on the tissue culture plate without any cotton sample.

### 3.7. SEM Analysis of the Bacterial Adhesion on Cotton Gauzes

**Figure 6:**
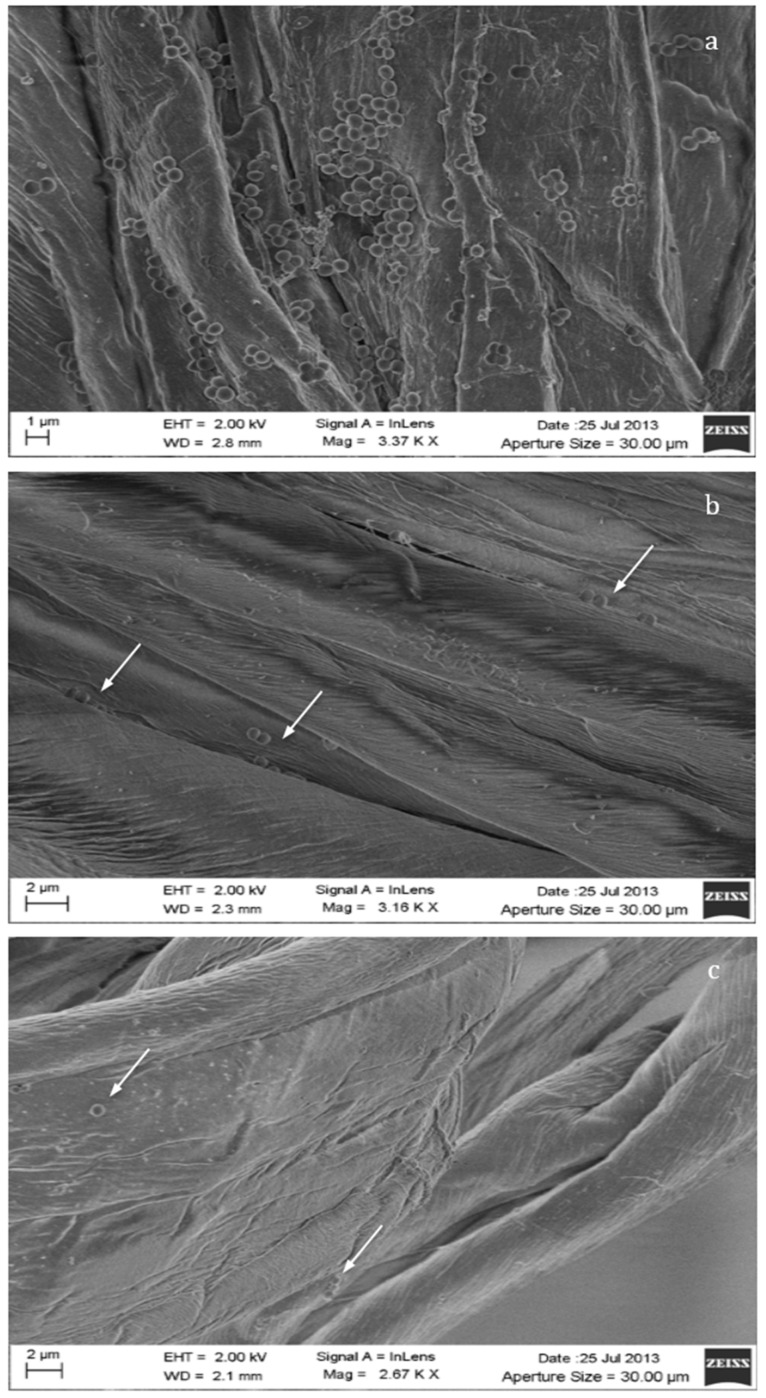
SEM analysis on bacterial cells adhered and proliferated on the cotton gauzes: (**a**) untreated gauze; (**b**) gauze treated with 0.5 wt/v % silver; (**c**) gauze treated with 4 wt/v % silver.

The efficacy of the silver coating in inhibiting the adhesion and the proliferation of bacterial biofilm on the cotton gauzes was analyzed through scanning electron microscopy. The SEM pictures reported in Figure 6a–c indicate the evident reduction in the adhesion of the bacterial cells to the fibers induced by the presence of silver. Indeed, a high number of *S. aureus* colonies can be observed on the neat cotton fibers (Figure 6a), whilst few isolated bacterial cells are visible on both the silver-treated samples (Figure 6b,c, arrows).

### 3.8. Cytotoxicity Test

**Figure 7:**
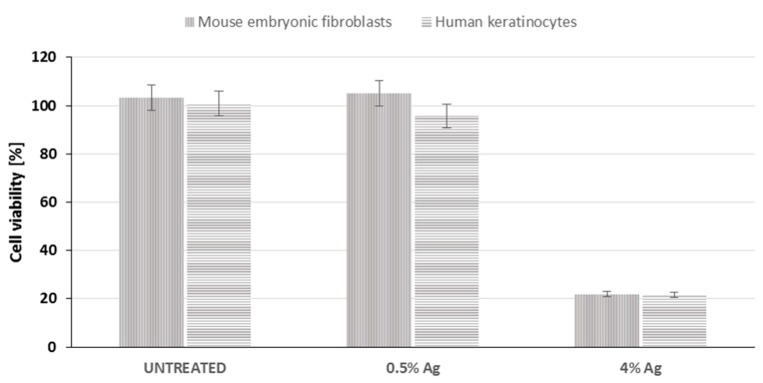
MTT assay for cytotoxicity evaluation.

Potential cytotoxicity associated to the presence of silver coating has been investigated by MTT assay through the extract method on murine fibroblasts 3T3 and human keratinocytes HaCaT. The results obtained are reported in Figure 7 as cell viability percentage, compared to cells cultured without any extract. The percentage of cell proliferation results were 103%, 105% and 21% in the presence of the extracts from untreated sample and sample treated with 0.5 wt/v % Ag and 4 wt/v % Ag respectively.

### 3.9. ICP-MS Analysis

**Figure 8:**
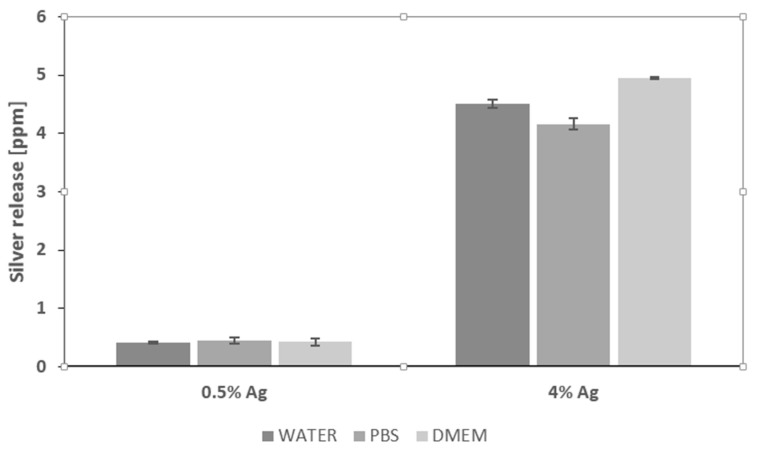
ICP-MS analysis performed in water, PBS and DMEM.

The release of silver in different media was calculated through ICP-MS analyses in static conditions, aiming to evaluate the stability of the silver coating and its possible interaction with biological fluids in terms of adhesion and cytotoxicity. The gauzes treated with 0.5 wt/v % released 0.409 ± 0.015, 0.449 ± 0.056 and 0.425 ± 0.055 ppm respectively in deionized water, in PBS and in DMEM. On the other hand, the gauzes treated with 4 wt/v % of silver released 4.505 ± 0.071, 4.160 ± 0.099 and 4.954 ± 0.014 ppm in the same media (Figure 8).

## 4. Discussion

The aim of this work was the development of silver-treated cotton gauzes and their evaluation for potential application as antibacterial dressings in wound management.

As defined in the literature, an ideal wound dressing should maintain a moist environment and oxygen permeation, should absorb excess exudates and prevent bacterial contaminations, and should be non-adherent to the wound and easily removable [24,25]. The devices developed in this work are intended to provide absorption due to the presence of cotton, and to prevent infections due to the presence of silver. However, they can also be proposed as an inherent part of a more complex device, where emollients and hydrogels can be added for improved hydration and comfort. Some wound dressings including hydrocolloids and/or other substances, have also been proposed for providing bioburden control, fast wound healing, ease of use and cost-effectiveness [26,27,28]. Moreover, today silver-containing dressings are widespread in the management of burn injury and acute and chronic wounds [28,29,30]. In addition to antimicrobial activity, silver dressings may modulate or reduce wound pain [31], and also an active role in wound healing has been associated to the presence of silver [32,33]. When compared to many wound dressings mainly based on nanocrystalline silver and silver compounds, the silver-modified gauzes presented in this work are characterized by some distinctive features related to both the nature of the materials and the production process adopted. Indeed, most of the wound dressings available today are obtained by synthetic materials, such as polyethylene, and are obtained through more complex production processes often involving the use of binders [34,35,36,37,38].

The main groups of silver dressings available on the market are dressings with sustained levels of silver release such as Acticoat, dressings with lower levels of silver release such as Actisorb and Aquacel, dressings with high concentration of silver at wound surface such as Contreet Foam, and dressings that release silver compounds rather than silver ions such as Urgotul [34].

In addition to the mechanism for silver release, these products are also different in terms of nature and composition. For example, Aquacel Ag is a hydrofiber-based wound dressing made of soft non-woven sodium carboxymethyl cellulose fibers integrated with ionic silver, while Urgotol Silver, developed from a lipido-colloid technology, is a non-adhesive and non-occlusive dressing made of polyester textile mesh impregnated with hydrocolloid particles and vaseline, where silver is incorporated as silver sulphate [39,40].

On the contrary, these devices are completely natural and, although the silver particles lack chemical bonds to link with natural fibers [37], an excellent adhesion of the coating to the substrate has been obtained without the use of any binder [19,20,21,22]. No complex evaporation system or expensive equipment are necessary in this silver deposition process, thus addressing an important aspect related to the scaling-up of the technology.

This paper mainly involves microbiology and cytotoxicity aspects related to the specific application of the material, aiming in particular to verify the efficacy of the silver treated wound dressings in preventing infection in skin injuries in terms of bacteria biofilm adhesion and proliferation. Thus, in the first part of the work, the silver coating was characterized through TGA and SEM analyses in order to verify the presence and distribution of the silver particles on the substrate; the second part is mainly dedicated to the biological investigation, in terms of antibacterial capability and biocompatibility. For the specific application of the textile materials object of this research work, the experimental samples were obtained by depositing cotton gauzes with 0.5 wt/v % silver solution and the results were discussed in comparison with untreated gauze and gauze treated with 4 wt/v % of silver as control samples. The presence of silver on the substrates was verified through TGA analysis and resulted different between the treated samples, as expected. Interestingly, the other results obtained by the different characterizations demonstrated that no significant differences occurred between the samples treated with 0.5 wt/v % Ag and 4 wt/v % Ag. SEM analysis (Figure 1a–d) demonstrated that 0.5 wt/v % of silver ensured a homogeneous distribution of silver nanoparticles on the cotton fibers. In order to verify and quantify the antibacterial capability of the samples, qualitative and quantitative tests, namely agar diffusion (Figure 2a–c) and bacterial enumeration tests (Figure 3), were performed on *S. aureus*, as a representative microorganism responsible for skin and wound infections. As is visible in Figure 2, both the silver concentrations exhibited inhibition areas to bacterial growth larger than 1 mm, and the bacterial enumeration tests also demonstrated an impressive reduction of the bacterial proliferation associated to samples treated with both 0.5 and 4 wt/v % silver (Figure 3). The effect of the silver coating in preventing bacteria biofilm adhesion and proliferation was also evaluated through different experiments aiming to analyze and quantify the bacterial adhesion on the cotton fibers. Textile materials can be a fertile ground for bacteria growth, and the wound site represents a particularly good environment for bacteria adhesion and proliferation [41]. The results obtained by bacterial biofilm enumeration tests demonstrated a high reduction of viability and proliferation of bacteria adhered to the surface of the device (Figure 4, Table 1), thus confirming the bacteriostatic and bactericidal effect of the silver coating. Indeed, the optical density measurements related to the total amount of bacteria demonstrated 1 log reduction between untreated and silver treated samples, while the evaluation of bacteria viability through the serial dilutions method indicated log reduction >3, thus confirming the bactericidal effect of deposited silver [42,43]. Moreover, fluorescence microscopy performed on bacteria stained on the multi-well plate after incubation with and without the samples (Figure 5) indicated that the number of bacteria was significantly reduced in presence of the silver coatings (Figure 5c,d), in comparison with the untreated sample (Figure 5b) and control (Figure 5a). As expected, a high concentration of bacteria organized in clusters of colonies was observed in control sample, *i.e.*, in case of only medium inoculated with bacteria. On the other hand, compared to control, the untreated sample (Figure 5b) resulted in a lower concentration of bacteria adhered on the multi-well plate, because of the partial adhesion of bacteria also on the gauze surface. At this purpose, SEM analysis was also performed on bacteria biofilm grown on the textile substrates (Figure 6a–c), confirming the effectiveness of the silver coatings in reducing the bacteria adhesion and proliferation on the gauze. Indeed, while a few isolated bacterial cells can be observed on the silver-treated samples (Figure 6b,c), a large number of bacterial colonies aggregated in bacterial communities can be observed on the untreated sample, indicating an initial step of biofilm growth (Figure 6a). The presence of silver coating successfully inhibited the bacteria growth, and significant differences were not observed between the silver concentrations tested. All the results indicated that the silver coating obtained by 0.5 wt/v % Ag solution was as effective as 4 wt/v % Ag against *S. aureus*, and that it successfully inhibited the bacterial colonization and biofilm formation on the dressing. In the graph reported in Figure 7, no significant differences can be observed in percentage of cell proliferation between untreated and 0.5 wt/v % Ag-treated samples. These data confirmed no cytotoxic effect of silver treated textile substrates and no skin irritation or hypoallergenicity effects through *in vivo* testing on selected patients [44,45,46,47]. Although the cell viability was reduced by the samples treated with 4 wt/v % silver, the highest values of silver release produced results that were still lower than the limit of cytotoxicity reported in the literature [48,49,50]. On the other hand, the presence of the silver coating on the cotton gauze treated with 0.5 wt/v % did not affect the cell viability. ICP-MS analysis performed in different media demonstrated the strong adhesion of the coating to the cotton substrate, thus also confirming that very low amounts of silver are released by the experimental samples even in simulated physiological conditions. The experiments were carried out in deionized water as control, in PBS in order to reproduce physiological conditions, and in DMEM in order to relate the data to cytotoxicity tests (Figure 8). As expected, samples treated with 4 wt/v % Ag released values of silver higher than samples treated with 0.5 wt/v %, and the results were consistent with the biological characterization. Moreover, the presence of a biological environment did not affect the silver release, thus confirming the stability of the coating and the perfect adhesion to the textile substrate.

## 5. Conclusions

The aim of this work was the development of effective and low-cost silver dressings for the prevention of bacterial infections in wound, burns and skin injuries. Conventional cotton gauzes extensively used in wound care for their absorbent properties and economic features were deposited with silver nanoparticles through the *in situ* photo-reduction of silver nitrate. A low content of silver (0.5 wt/v %) was adopted and tested in comparison with a higher silver percentage (4 wt/v %) and an untreated sample. TGA and SEM analysis demonstrated the presence of nanoparticles and their good distribution on the treated samples. The microbiological characterizations demonstrated that the gauze treated with 0.5 wt/v % of silver was as effective as that treated with 4 wt/v % of silver against bacteria in terms of viability and biofilm adhesion and growth. Due to the good antimicrobial properties and biocompatibility demonstrated, the textile materials developed can be considered a promising alternative to conventional wound dressings, with clear advantages in terms of prevention of infections and costs.

## Figures and Tables

**Table 1:** Quantification of bacterial biofilm on cotton gauzes through serial dilution method.

Sample	CFU/mL	Log Reduction
Untreated	1.62 × 10^6^	–
0.5% Ag	5.27 × 10^2^	3.49
4% Ag	5.00 × 10	4.51

## Abbreviations

The following abbreviations are used in this manuscript:

SEM	Scanning Electron Microscopy
TGA	Thermo-Gravimetric Analysis
MTT	3-[4,5-dimethylthiazol-2-yl]-2,5-diphenyltetrazolium bromide colorimetric assay
ICP-MS	Inductively Coupled Plasma Mass Spectrometry
